# Real-Time Vision-Based Stiffness Mapping †

**DOI:** 10.3390/s18051347

**Published:** 2018-04-26

**Authors:** Angela Faragasso, João Bimbo, Agostino Stilli, Helge Arne Wurdemann, Kaspar Althoefer, Hajime Asama

**Affiliations:** 1 Department of Precision Engineering, School of Engineering, The University of Tokyo, Hongo 7-3-1, Bunkyo-ku, Tokyo 113-8656, Japan; asama@robot.t.u-tokyo.ac.jp; 2 Istituto Italiano di Tecnologia (IIT), Via Morego, 30 16163 Genova, Italy; joao.bimbo@iit.it; 3 Department of Computer Science, University College London, London WC1E 6BT, UK; A.Stilli@cs.ucl.ac.uk; 4 Department of Mechanical Engineering, University College London, London WC1E 7JE, UK; h.wurdemann@ucl.ac.uk; 5 Centre for Advanced Robotics at Queen Mary (ARQ), Faculty of Science & Engineering, Queen Mary University of London, Mile End Road, London E1 4NS, UK; k.althoefer@qmul.ac.uk

**Keywords:** stiffness sensor, soft tissue characterization, hand-held probe, medical examination, palpation

## Abstract

This paper presents new findings concerning a hand-held stiffness probe for the medical diagnosis of abnormalities during palpation of soft-tissue. Palpation is recognized by the medical community as an essential and low-cost method to detect and diagnose disease in soft-tissue. However, differences are often subtle and clinicians need to train for many years before they can conduct a reliable diagnosis. The probe presented here fills this gap providing a means to easily obtain stiffness values of soft tissue during a palpation procedure. Our stiffness sensor is equipped with a multi degree of freedom (DoF) Aurora magnetic tracker, allowing us to track and record the 3D position of the probe whilst examining a tissue area, and generate a 3D stiffness map in real-time. The stiffness probe was integrated in a robotic arm and tested in an artificial environment representing a good model of soft tissue organs; the results show that the sensor can accurately measure and map the stiffness of a silicon phantom embedded with areas of varying stiffness.

## 1. Introduction

Palpation is frequently used in medical practice and plays an important role in physical diagnosis [1]. A palpation procedure involves the examination of the patient by means of direct physical contact. The physical investigation is usually performed manually—clinicians touch organs or body parts with their fingers in order to evaluate the stiffness distribution in the examined body part. In most cases, the clinician aims to distinguish between areas of higher and lower stiffness, since these stiffness variations can give an indication of disease. The diagnosis is a function of kinaesthetic sensations: the indentation depth (i.e., how much the soft surface deforms) and the experienced reaction force. Palpation is one of the most effective methods used by medical experts to determine pathologies [2]. When practitioners attempt to detect tumours in some specific area of the human body, they discriminate between healthy regions and regions of abnormality, as indicated by stiffness differences, as the stiffness of a tumour is typically higher than that of the surrounding healthy tissue [3]. For instance, palpation-based breast examination should be conducted in a methodical way and performed over the entire breast. Suspicious breast lesions are hard and fixed rather than movable, the latter being an indicator of healthy tissue. Furthermore, it is noted that cancerous tumours are usually not tender, whilst benign lesions are more likely to be round, elastic or firm, movable, and well-defined [4].

It is reported that the sensitivity of palpation procedures, i.e., the clinician’s ability to correctly identify a tumour, is subjective and highly dependent upon the skill and experience of the practitioner—a skill often difficult to master [1,5,6]. Inadequate clinician training may contribute to a wide range of differences in diagnosis results based on such physical examination. During the last two decades, researchers have developed several instruments able to mimic the traditional manual palpation techniques that may be reliable, but are often difficult to use in a clinical setting [7]. It is noted that a system able to provide feedback to medical experts’ real-time quantitative measurements of soft tissue properties, force and stiffness is still missing and highly desirable [8,9]. Such system could be used for different purposes, e.g., to train early-career clinicians to reliably distinguish between the stiffness values of different types of tissue, a task which currently is performed and limited by medical simulators [5], to have quantitative measurements in tele-palpation tasks and even as a portable breast self-examination (BSE) device that can be used by women at home in order to detect subtle changes in breast tissue. The quantification of stiffness changes during BSE may provide important diagnostic information and aid in the early detection of minor abnormalities before they become serious.

**Figure 1:**
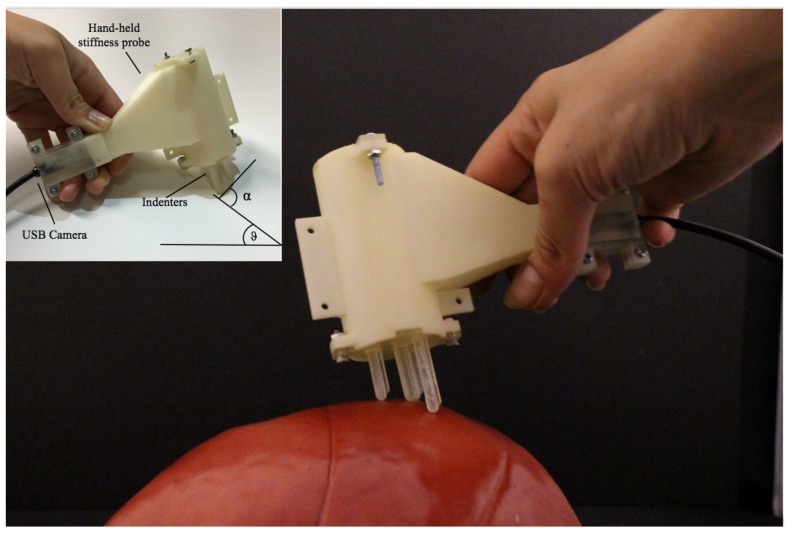
Multi-axis stiffness sensor interacting with a soft surface. The definition of the angles is represented in the upper left part of the image.

In this paper, we propose a vision-based multi-directional stiffness sensor, as shown in Figure 1. The paper is organised as follows: Section 2 reviews the current state of the art in stiffness sensing technologies. The mechanical design and sensing principle of the proposed prototype are described in Section 3. The mathematical model derived and the optimisation algorithm are presented in Section 4. The derivation of the real-time mapping is described in Section 5. The experimental results are reported in Section 6. Conclusions are drawn in Section 7.

## 2. Background

Early diagnosis, during the first stage of cancer development, leads to a precocious therapeutic strategy, with high chances of recovery or prolongation of the patient’s life expectancy. Changes in tissue stiffness may be an indication that cancer is present and thus one of the points of interest within the robotics and haptics research community has been the development of methods for tissue elasticity measurement. Therefore, starting from the early 1990s, in order to enhance the outcome of physical examinations, different instruments and technologies have been developed and explored. Indentation is one of the most exploited diagnostic techniques used to measure reaction forces as well as stiffness of a probed tissue [10]. Generally, indentation systems employ an indenter, which is used to compress the soft tissue, and sensing technologies that are used to compute the reaction force and the indentation depth. Combining the indentation depth and the reaction force the soft tissue stiffness can be estimated [7]. Linear elastic modelling of soft tissues is the most widely used approach [11]. This model assumes that the tissue obeys the generalized Hooke’s law; thus, similarly to springs, it is able to resume its configuration after the application of a force. Fung et al. [12] demonstrate that if the displacement induced in the soft tissue is small, the physical response can be modelled using the linear approximation, but, as the displacement increases, the linear elastic model becomes inaccurate. A Tactile Tumour Detector (TTD) for breast examination is presented in [13]. The main parts of the tactile sensing instrument are a tactile probe, an electrical circuit, a data processor and a tactile display. This device is able to detect abnormal objects embedded in soft tissue, but it works only with specific simulated artificial models of the anatomical area and cannot be used in real time. Most of the developed instruments work only if the orientation of the sensor does not change during the procedure [14]. Despite all the research efforts, problems related to miniaturization, ergonomics, integration in the medical setup, complexity and reproducibility have to be solved if these instruments are to be used in medical diagnosis. Moreover, to be approved by the medical community, the new systems should be ergonomic and intuitive. For instance, a medical palpation device should include computerised algorithms able to interpret the information acquired during the examination and to develop an intelligible representation of it that has to be conveyed to the clinicians. An explicit and intuitive representation of the measured soft tissue stiffness is obtained by a real-time colour codification. Hence, the stiffness distribution of anatomical surfaces can be stored during the medical examination in a colour-coded stiffness map. This map will help the clinicians to assess the stiffness distribution of the overall examined area, thus to easily detect abnormalities. In order to have multi-axial capabilities, researchers have been exploring rolling indentation mechanisms that enable the scanning and computation of forces on a surface rather than at a single point. For instance, Liu et al. [15,16] proposed a rolling indentation device that can slide on the soft tissues and compute the reaction force as well as identify tissue stiffness distribution in minimally invasive procedures. Optical fibres are employed to sense both axial force and indentation depth and are used to estimate the stiffness distribution of the soft surface. However, this sensor employs the use of a commercial force sensors and hence is not compatible with the medical setup. In addition, it is sensitive to the light changing and bending of the fibres, and requires a laborious calibration process and pre-registration of the soft surface.

**Figure 2:**
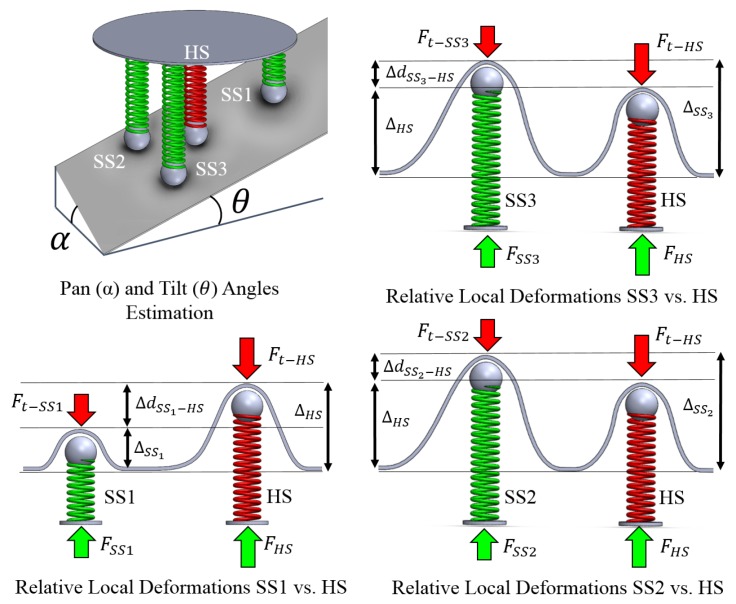
Operating mechanical principle. Contact between the soft surface and the sensor showing the interacting forces and the differential force between the linear model with an embedded hard spring (HS) and the three modules with embedded softer springs (SS1, SS2, SS3).

In previous works, the authors proposed a single axis force sensor device using a vision system to “view” the deformation of the sensing element for minimally invasive surgery (MIS) [17]. The sensing principle is based on the tracking of a feature in an image that is correlated to the compression of a spring. To overcome the limitations of the first prototype, a new sensing concept capable of estimating tissue stiffness values has been proposed in [18,19]. The proposed sensing mechanism employs the fabrication of a low-cost passive device that is fully 3D printed, does not require any troublesome calibration process and does not use any electronics. This work is extended from [19] where we proposed a multi-directional stiffness probe embedding four springs of different elasticity. The operating mechanical principle of the device is shown in Figure 2. In particular, this paper has the following contributions:

1. Further investigation of the influence of the inclination of the probe on the computation of the stiffness.
2. Generation of a colour-coded stiffness map using a magnetic tracking mechanism.
3. Evaluation of the sensor performance in robotic palpation.

## 3. Stiffness Sensor Methodology and Design

### 3.1. Mechanical Design

The proposed stiffness probe is composed of four indenters, each of which is connected to a spring and a spherical feature, and a standard USB camera tightly assembled in a 3D printed shell. The USB camera has an outer diameter of 7mm, a resolution of 640×480 and a frame rate of 30fps. Three of the springs have the same spring constant of 0.05N/mm; the fourth has been chosen stiffer with a spring constant of 0.25N/mm. The softer springs are placed on the vertices of a triangle and the stiffer one on its barycentre. During interaction with a soft object, the indenters slide over their rods leading to a compression of the correspondent spring, which generates, in turn, a movement of the related spherical features. The relationship between the movements of the spring, the related indenter and the spherical feature is regulated by the elasticity of the springs: i.e., contact with an external soft surface will produce a bigger compression of the three softer springs than of the stiffer one. Consequently, the indenters associated with the softer springs will slide more than the one associated to the stiffer, generating different displacements of the spherical features along the horizontal axis. The spatial resolution of the probe is related to the area of the triangle drawn by connecting the tip of the three indenters related to the soft springs; hence, the smallest detectable stiff object in the soft tissue should at least have an area of 131.8mm. In addition, a consistent reduction of the distance can cause interferences between the indenters and results in wrong estimations.

### 3.2. Image Processing Algorithm

The image processing algorithm consists mainly of the detection and tracking of coloured spheres in an image. In order to compute the intrinsic parameters of the camera and eliminate distortions of the lens, the camera calibration toolbox in the robot operating system (ROS), was used. The algorithm involves different steps: image-filtering in the HSV (hue, saturation and value) color space to detect the coloured feature; in the obtained greyscale image, four Regions of Interest (RoIs) are selected. Each RoI contains a section of the image that is related to the full range of motion of one spherical feature. In each RoI, the morphological operators *dilate* and *erode* are applied to eliminate the bias and reduce the noise. The sliding movements of the feature’s centroid along the horizontal axis are then tracked using the image moment. A Kalman filter is applied to robustly track the features, and the position of each centroid is mapped in a fixed reference system with its origin at the sensor. The mapping between the movements of the feature’s centroid in the image, in pixels, and the sliding of the correspondent indenter in mm, depends on the camera’s resolution and has been computed experimentally.

### 3.3. Spring-Indenter-Feature Analogy

**Figure 3:**
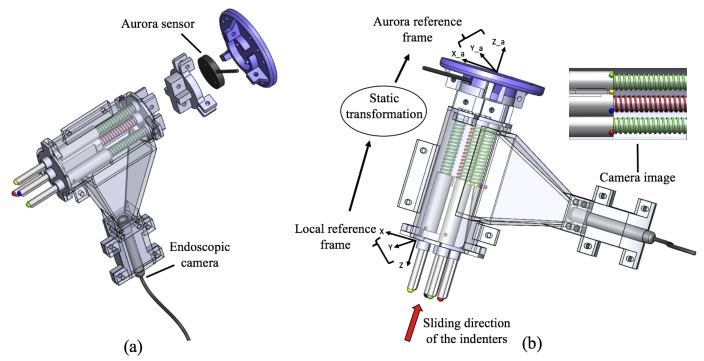
Stiffness probe integrated with a commercially available tracking system: (**a**) Exploded view of the sensor showing the position of the Aurora tracker. (**b**) Schematic representation of the sensor’s working principle: the interaction with external objects generates the sliding of the indenters, hence, the visual features change their positions in the camera’s image. The correlation between the indenters and the features allows to measure the new positions of the indenters in the local reference frame. A static transformation maps the new positions of the indenters from the local frame into the tracker frame.

A local reference system was chosen on the stiffness probe as shown in Figure 3b. The image plane is parallel to the *x*–*z* plane of the local reference system on the probe and the position of the indenters is expressed in this frame. As the springs are compressed or decompressed, the indenters are sliding, changing their *z*-position in the local reference frame; consequently, the spherical features are moving, changing their *x*-position in the image plane. The relationship between the springs, the indenter and the spherical features allow mapping the variation in position of the centroids, to the change in depth of the indenters.

## 4. Methodology

### 4.1. Modelling Soft Tissue Properties

The mapping between the position of the spherical features in the image and the position of the indenters is used to characterise the interaction between our device and the probed tissue. The sliding motion of the three indenters placed on the vertices of the triangle make use of the same type of spring, while the indenter in the centre is connected to a spring with a higher spring constant, thus it moves less during the interaction with the soft tissue. The stiffness of the surface in contact can be computed using the forces applied by the harder indenter placed in the barycentre of the triangle and the three softer indenters [18]. In our previous study, we proposed a uniaxial stiffness sensor; we further developed our system to make the stiffness computation independent of the pan and tilt angles with respect to the tissue surface.

Seven parameters are sufficient to fully characterise a palpation procedure with the proposed stiffness probe: the stiffness of the soft tissue, $K_{t}$, the four palpation depths of the soft tissue caused by the interaction with the four indenters, the pan angle θ and the tilt angle α. A system of nonlinear equations, F(x)=0, can be used to express the geometrical relation between the seven unknown parameters in the local reference frame described in Section 3.3, where:

(1) $$F\left(x\right)=\left\{\begin{array}{c} dx_{1}\times K_{s}-K_{t}\times dt_{1}\\ dx_{2}\times K_{h}-K_{t}\times dt_{2}\\ dx_{3}\times K_{s}-K_{t}\times dt_{3}\\ dx_{4}\times K_{s}-K_{t}\times dt_{4}\\ dx_{2}+dt_{2}-dx_{1}-dt_{1}+\tan\left(\theta \right)\times d_{12x}+\tan\left(\alpha \right)\times d_{12y}\\ dx_{3}+dt_{3}-dx_{1}-dt_{1}+\tan\left(\theta \right)\times d_{13x}+\tan\left(\alpha \right)\times d_{13y}\\ dx_{4}+dt_{4}-dx_{1}-dt_{1}+\tan\left(\theta \right)\times d_{14x}+\tan\left(\alpha \right)\times d_{14y}. \end{array}\right.$$

In Equation (1), $dx_{i}$ (i=1...4) represents the position of the *i*th sphere, $d_{ijx}$ and $d_{ijy}$ (i,j=1...4) with i≠j represents the distance between the *i*th and *j*th sphere in the reference system of the sensor. $K_{s}$ and $K_{h}$ are the known spring constants of the soft and hard springs, respectively. The seven unknown parameters are: the pan angle θ, the tilt angle α, the displacement of the soft tissue in the points of contact, $dt_{i}$ with (i=1...4) and the stiffness of the soft surface $K_{t}$, and are collected in the vector of the problem’s unknowns, $x={\left[K_{t},dt_{1},dt_{2},dt_{3},dt_{4},\alpha ,\theta \right]}^{T}$. Solving the system of nonlinear equations F(x), in Equation (1), involves finding a solution such that every equation in the nonlinear system is equal to zero, i.e., find a vector $x^{★}$ such that $F(x^{★})=0$. The major algorithms used for solving nonlinear equations proceed by minimizing a sum of squares of the nonlinear equations, which is equivalent to an unconstrained nonlinear least squares problem.

### 4.2. Soft Tissue Characterisation

The Levenberg–Marquardt algorithm (LMA) is applied here to solve Equation (1). This algorithm is an iterative optimisation technique for solving nonlinear systems of equations and least squares problems. To solve the convergence issues of the iterative process, the LMA combines the advantages of the gradient-descent and the Gaussian–Newton methods [20]. The LMA provides the best compromises between complexity, stability and speed [20]. The optimization method is used to find at each iteration the update rule of the vector x:

(2) $$x_{k+1}=x_{k}-{\left(H\left(x_{k}\right)+\lambda_{k}diag\left(H\left(x_{k}\right)\right)\right)}^{†}J{\left(x_{k}\right)}^{T}F\left(x_{k}\right),$$

where $J(x_{k})$ is the Jacobian matrix of F(x) evaluated at $x_{k}$, $H\left(x_{k}\right)\approx J{\left(x_{k}\right)}^{T}J\left(x_{k}\right)$ is an approximation of the Hessian matrix and $\lambda_{k}$ represents the non-negative damping factor that is adjusted at each iteration to interpolate between the gradient descent and Newton’s method. Using high values for λ favours gradient descent, whereas using lower values favours Newton’s method. The damping factor λ is increased by a factor of μ, if $∥F(x_{k+1})∥$ is greater than $∥F(x_{k})∥$ and decreases by a factor of μ otherwise. The proposed method converges when the cost function, $g=∥F\left(x_{k+1}\right)-F\left(x_{k}\right)∥$, is less than a chosen threshold ε. Then, the current vector $x_{k+1}$ is returned as the best-fit solution $x^{*}$.

In order to be able to find a solution to the unknown parameters and to compute the stiffness in real time, in the implemented algorithm, the threshold ε and the maximum number of iterations $k_{max}$ are set 0.0001 and 100, respectively. The final threshold value was found through a trial-and-error approach. Using this threshold, the algorithm typically needs less than seven iterations to find a solution with high accuracy. The value of μ used to adjust the damping parameter at each iteration is equal to 10. Algorithm 1 illustrates the procedure used to solve the system presented in Equation (1).

**Algorithm 1 **Iterative procedure solving Equations (1): LMA

**Input:**
  *F*, the cost function  $x_{0}$, initial solution
**Output:**
  $x^{★}$, local minimum of *F* 1:
**Begin**
 2:  k←100 3:  λ←20 4:  ε←0.0001 5:  μ←10 6:  $x_{k}\leftarrow x_{0}$ 7:  **while** g>ε**and**$k<k_{max}$ **do** 8:    $x_{k+1}\leftarrow x_{k}+\lambda_{k}$ 9:    **if** $F\left(x_{k+1}\right)<F\left(x_{k}\right)$ **then** 10:      $x_{k}\leftarrow x_{k+1}$ 11:       $\lambda \leftarrow \frac{\lambda }{\mu }$ 12:    **else** 13:      λ←λμ 14:    **end if** 15:    k←k+1 16:  **end while** 17:  **return** $x_{k}$ 18:
**End**



## 5. Real-Time Stiffness Mapping

In order to map the stiffness of the soft tissues in real time when performing palpation with a medical instrument, the pose of the device needs to be measured and recorded. Different technologies can be used to assess the 3D position of an object in real time, e.g., magnetic, optical and visual motion tracking systems. Moreover, in medical systems employing robotic platforms, i.e., teleoperation, the device is attached to the end-effector of a robot arm. Thus, the kinematic of the robot is used to retrieve the pose of the tool attached to its end-effector.

The multi-directional stiffness probe has been integrated with a commercially available tracking system and used to evaluate the stiffness of soft tissues. Hence, the pose of the device is used to record a colour-coded stiffness map in real-time. In addition, to evaluate the performance of the proposed sensory mechanism in robot assisted technologies and obtain a fine map, the device has also been fixed to the tip of a robotic arm, thus the kinematic of the robot is used to map and record the measured stiffness. Three of the indenters are allocated in the vertex of a triangle and one on its barycentre. The mapping of the positions and orientations of the indenters is used to visualise and record a triangle in a global colour-coded stiffness map. The colour of the visualised triangle is a function of the measured stiffness, i.e., light colours are associated with low values of the soft tissue stiffness and dark colours to higher values. The association of the stiffness of the examined area to the displayed colour in the map allows for the easy determination of the variation of the mechanical properties of the soft tissue. Furthermore, after the examination of the anatomical area, the colour-coded stiffness map can be used to evaluate the stiffness distribution on the entire surface.

### 5.1. Pose Estimation with a Magnetic Motion Tracking System

One Aurora magnetic tracker by Northern Digital Incorporated (Waterloo, ON, Canada) is fixed to an allocated position on the stiffness probe, as shown in Figure 3a. Thus, the position and orientation of the probe can be tracked and recorded whilst examining a tissue area. The developed system does not use any electronic or metal sources, so there is no risk of affecting the performances of the electromagnetic tracking system. The pose of each indenter in a resting condition is expressed in a local reference frame that is fixed on the stiffness probe as shown in Figure 3b. These positions are jointly mirrored to specific positions of the visual features in the image. A static transformation from the local reference frame into the Aurora tracker frame ^a^$T_{p}$ is used to record the global positions and the orientation of the indenters:

(3) $${}^{t}T_{p}={}^{t}T_{a}{}^{a}T_{p},$$

where ^t^$T_{a}$ expresses the position of the tracker and ^a^$T_{p}$ is the translation matrix, along the *z*-axis, between the tracker frame and the frame with origin on the stiffness probe.

### 5.2. Pose Estimation with a Fixed Base Robot Arm

**Figure 4:**
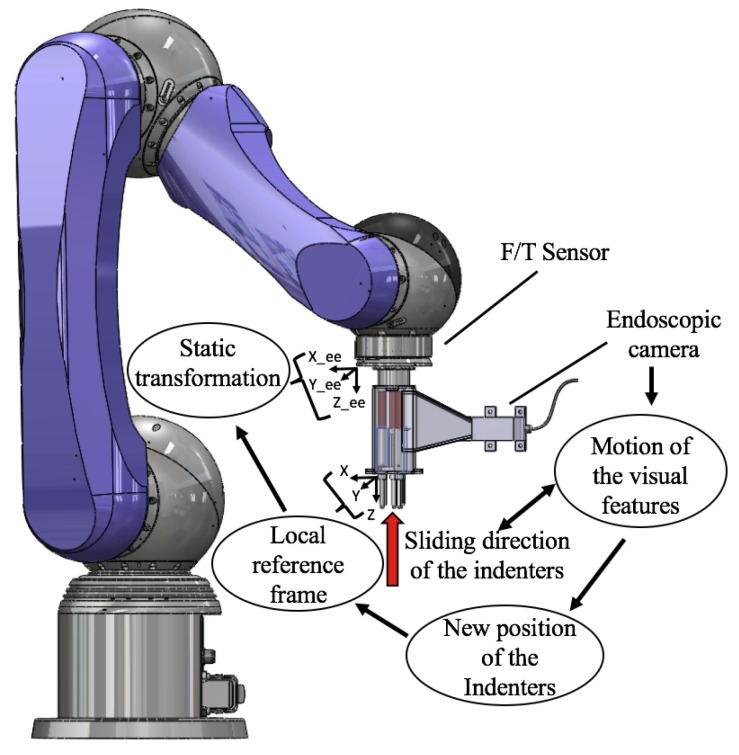
Stiffness probe fixed at the tip of the robotic arm: the interaction with external objects generates the sliding of the indenters. Hence, the visual features change their positions in the camera’s image. The correlation between the indenters and the features allows for measuring the new positions of the indenters in the local reference frame. A static transformation maps the new positions of the indenters from the local frame into the robot kinematic chain.

To evaluate the performance of the vision-based stiffness sensing mechanism in medical robotics applications, the stiffness probe is fixed to the end-effector of the Lightweight 6 degrees of freedom (DOF) robot arm LWA 4P by SCHUNK GmbH & Co. KG (Lauffen am Neckar, Germany) . A multi-axis ATI force/torque sensor by ATI Technologies Inc. (Markham, ON, Canada), is attached to the end-effector of the robotic arm and the stiffness sensor is fixed to the front plate to palpate silicone phantoms as shown in Figure 4. The kinematics of the robotic arm are used to compute the pose of the stiffness probe while recording the reaction forces measured by the force/torque sensor in real time.

Given the six joint values, [$\theta_{1}$,..., $\theta_{6}$ ], and the Denavit–Hartenberg (DH) parameters, the pose of the end-effector is fully determined by the homogeneous transformation matrix ^0^$T_{6}$. Consequently, the position of the local frame with origin on the stiffness probe is obtained by multiplying the homogeneous matrix for the translation matrix ^6^$T_{p}$, which expresses a translation along the *z*-axis. The transformation matrix that expresses the local frame with origin on the stiffness probe into the base frame is:

(4) $${}^{0}T_{p}={}^{0}T_{6}{}^{6}T_{p}.$$

## 6. Experimental Results

Different experiments were carried out to analyse the performance of the proposed stiffness probe. The proposed algorithm runs at the same rate of the USB camera embedded in the device, i.e., 60Hz. The first experiment was conducted in order to verify the accuracy of the probe in measuring the stiffness of different artificial stiffness samples. In the second experiment, manual palpation of silicone phantoms was performed to evaluate the ability of the sensor in distinguishing materials that present different stiffness and study the effect of the probe’s orientation on the the computation of the stiffness of the probed material. In the last two experiments, a real-time stiffness map of a silicone phantom with areas of varying stiffness has been generated using a magnetic tracking system and a robotic arm. The benchmarking stiffness value of the artificial and silicone samples has been computed using an ATI Nano-17 6 DOF force/torque sensor (SI-12-0.12, resolution 0.003 N with a 16-bit data acquisition card). All the software has been developed using ROS. The computation of the stiffness is performed at the same rate of the USB camera embedded in the device, i.e., 60Hz. The ROS node that updates the color-coded stiffness map runs at 40Hz, i.e., a new triangle is added to the map every 25ms. The following subsections will explain the experimental tests in more detail.

### 6.1. Evaluation Test with Stiffness Samples

**Figure 5:**
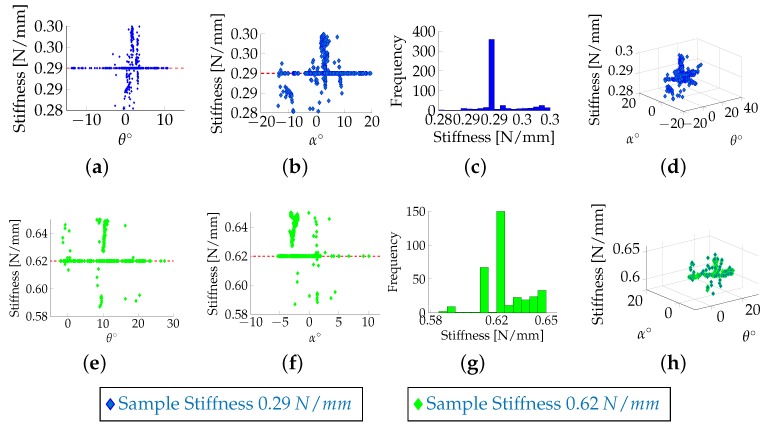
Manual tests for the artificial stiffness samples with spring constant of 0.29 N/mm and 0.62 N/mm: Correlation between the measured stiffness and the orientation of the hand-held probe, which is defined by the pan angle θ (**a**,**e**) and the tilt angle α (**b**,**f**); Distribution of the stiffness during the experiments (**c**,**g**); The stiffness variation in function of the two angles (**d**,**h**). The mean of the stiffness and the standard deviation are 0.29 and 0.01 for the first sample, and 0.68 and 0.08 for the second.

The linear elastic model was used here to mimic human soft tissue. In this model, the tissue is considered as a homogeneous, linear elastic material; the stress/strain relationship is assumed to be linear too. Although biological tissues are much more complex, this behaviour was found coherent for a relative strain under 10 to 15% [12]. Under this assumption, the material properties can be described using Hooke’s law. The complexity of the model depends on the deformation range: under geometrical linearity, (i.e., small deformations), the Green–Lagrange equation that relates stress and strain tensor is linearised by neglecting the second order term [21]. Therefore, in this model, a series of parallel springs [22] can be used to represent the soft tissue. Artificial stiffness samples, each embedding four parallel springs in series, have been designed in [19]. Each sample contains four rods with self-centring shaped heads and their movements are related to four linear springs with identical spring constants. To evaluate the multi-directional capability of the system, the stiffness probe was manually pushed against the artificial stiffness samples. The experimental results demonstrate that, for angles within the interval $\left[+30^{\circ },-30^{\circ }\right]$, the computation of the stiffness does not depend on the orientation at which the device approaches the samples: there is no correlation between the computation of the stiffness and the pan and tilt angles of the probe, as shown in Figure 5. It can be noticed that there is a consistent variation of the system at a zero angle, which is due to the mechanical latency, e.g., the time needed for the indenters to reach the minimum distance between them that generates a valuable displacement, used for evaluation of the stiffness, between the relative visual features in the image. Moreover, the difference in force between the indenter associated with the stiff spring and the one associated with the soft springs, which influences the computation of the stiffness, increases with the softness of the contact surface.

### 6.2. Stiffness Mapping of Silicone Phantoms

#### 6.2.1. Manual Palpation

**Figure 6:**
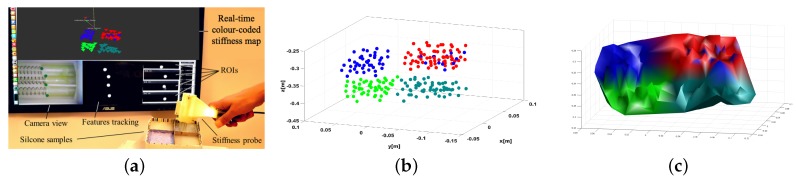
Stiffness mapping of homogeneous silicone phantoms: (**a**) the experimental setup; The discrimination between the different stiffness values of the silicone samples is visible in the coloured points in (**b**); The post-processed stiffness map is illustrated in (**c**).

The hand-held stiffness probe is equipped with the Aurora reference disk tracker, as shown in Figure 6a, and used to palpate silicone phantoms presenting different stiffness values while at the same time visualising and recording a colour-coded stiffness map. Silicone is a nonlinear elastic material. For small displacements, however, the response is approximately linear [12,15]. The material used are: Oomoo${}^{®}$ 30A, Dragon Skin${}^{®}$ 20A, Ecoflex${}^{®}$00-50 and Ecoflex${}^{®}$00-10 by Smooth-On, Inc. (Macungie, Pennsylvania, USA). The test results in Figure 6b,c show that our sensor is able to successfully distinguish materials with different stiffness levels even if the difference is relatively small as for example when comparing Dragon Skin${}^{®}$ 20A to Oodomo${}^{®}$ 30A—a scenario in which manual finger palpation fails. The values associated with those two materials are represented in blue and red in the post-processed Figure 6b,c. In particular, Figure 6b represents the position of the barycentre associated with the triangles in the reference frame of the magnetic tracker, which were computed in real-time during the tests. Figure 6c is the three-dimensional surface plot.

#### 6.2.2. Robotic Palpation

Evaluation tests have been performed using the SCHUNK Lightweight Arm LWA 4P, as shown in Figure 4. A multi-axis ATI force/torque sensor was attached to the end-effector of the robotic arm and the stiffness sensor was fixed to the front plate to palpate silicone phantoms. The forward kinematics of the robotic arm were used to compute the pose of the stiffness probe while the force/torque sensor recorded the reaction forces in real time. The silicone phantom used in this test is made of Ecoflex${}^{®}$00-50 by Smooth-On, a material which is similar in its mechanical behaviour to the human skin. The mathematical function that directly expresses forces measured in terms of the probe’s displacement is derived using a Matlab Curve Fitting Tool, R2016b by MathWorks, Inc., Natick, MA, USA, where the best fit to the data points is obtained using the linear function in Equation (5):

(5) $$F\left(x\right)=p_{1}\cdot x.$$

The value of the parameter found was $p_{1}=1.2\;N/\mathrm{mm}$. The fit has a prediction interval of 96% and its root-mean-square error (RMSE) is 0.028.

**Figure 7:**
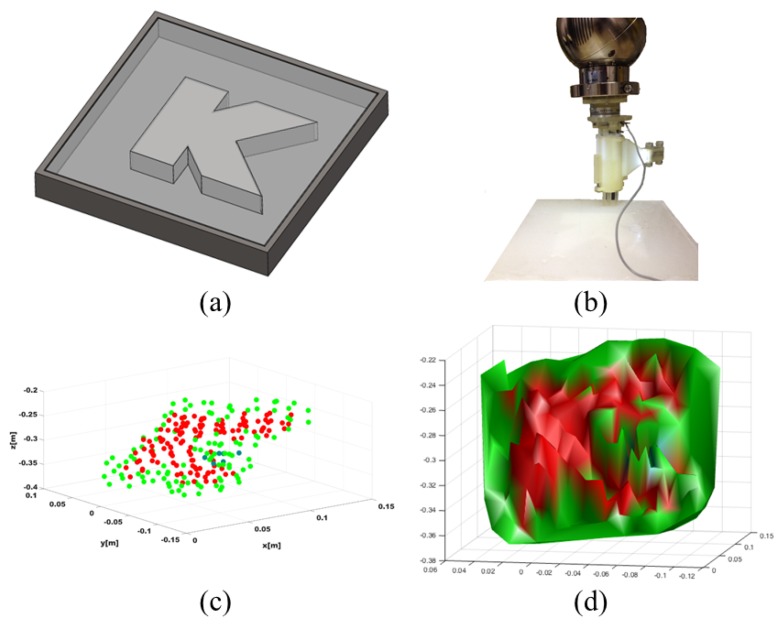
Computation of the stiffness map: CAD model of the phantom mould (**a**); Experimental setup (**b**); Post-processed map and generated surface are shown in (**c**,**d**). The stiffness of the track (red) is successfully distinguished from the surrounding silicone (green).

A customised phantom presenting areas of different stiffness values as shown in Figure 7a has been used to validate the ability of the proposed system in mapping the phantom’s stiffness in real time. The phantom mould has an embedded K-shaped track, which is etched (2mm depth) on the flat acrylonitrile butadiene styrene (ABS) surface, and has been filled with Ecoflex${}^{®}$00-10 by Smooth-On. The experimental rig and the results of the tests are shown in Figure 7. The final stiffness map is shown in Figure 7c. Using the stiffness probe with the robotic arm, it is possible to capture the difference in stiffness between the embedded silicone and the K-shaped track as shown in Figure 7c,d and obtain a fine map. For these reasons, our sensing mechanism has high potential in applications where clinicians need to distinguish between soft and stiff tissue areas for the detection of abnormalities.

## 7. Discussion

In this paper, further investigation on the multi-directional capability of the hand-held stiffness probe for medical palpation developed in our previous work [19] has been proposed. The stiffness probe has also been used to generate a global colour-coded stiffness map. Experimental tests with several silicone phantoms showed the accuracy of the sensing device in distinguishing between materials presenting different stiffness independently of the orientation at which palpation is performed. The pose of the probe is estimated through both a magnetic tracker and a fixed base robot arm of known kinematics. Results proved the effectiveness of the stiffness probe in both manual and robotics palpations. The system provides an intuitive user interface for real-time stiffness mapping of the stiffness distribution of anatomical surfaces. The colour-coded stiffness map gives a clear-cut representation of the stiffness of the examined area. Furthermore, it can be used to evaluate and record the stiffness distribution of all the surface. If used for medical diagnosis, it can help clinicians to obtain valuable information about the presence of a mass inside a soft tissue organ. That being so, it can potentially be used for tumour localisation. A customised phantom, embedding a stiffer track, was used to test the ability of the probe in detecting tumours. Results demonstrate the accuracy of the sensor in discerning between different materials embedded in the same phantom; thus, this device can be potentially used for medical diagnosis. Future developments of the system will consider an in-depth evaluation involving different soft materials, evaluation of the nonlinearity of the soft tissue as well as the integration of our sensing concept with an endoscopic camera and other surgical tools for MIS.

References
